# Diseases of Children

**Published:** 1894-07-28

**Authors:** 


### Medical Progress.

### DISEASES OF CHILDREN.

**Tuberculous Disease.**—At a recent meeting of the Medical Society of London, Dr. Walter Carr[11] read a paper on the starting points of *tuberculous disease in children*, based on the post-mortem evidence in 120 cases. He found that in 82 cases the disease was more or less generalised, in 13 the glands alone were implicated, in 13 the lesion was still limited to one organ (death having occurred from some other cause), and in only 12 had a localised tuberculous process been the actual cause of death. The starting point of the disease in the majority of cases is in the glands, and the thoracic glands are more frequently affected than the mesenteric. Hence he concludes that, although infection through milk does occur, yet infection through the air is more frequent and more important. As regards the prevention of tubercular disease, he lays stress on increasing the resistent powers of the tissues to the entrance of the tubercle bacilli, and more especially on strengthening the mucous membranes in rachitic subjects or those convalescent from fevers. Dr. Sidney Martin did not admit Dr. Carr's statistics regarding infection through the intestine, and pointed out that the presence or absence of a local lesion depended on the amount of the poison absorbed. A small dose might give rise to no visible primary lesion, while a larger dose usually did. The manner in which the bronchial glands become affected by tubercular disease is discussed by Dr. H. Neumann.[12] The infective material is conveyed either by hereditary transmission or by contagion. The former he considers to be very unusual, while the latter will explain many facts supplied by clinical experience. The diagnosis of tubercular bronchial glands is difficult, although in many cases possible. Stress is laid on the presence of symptoms of compression of the bronchi or adjacent nerves, on dulness over the anterior mediastinum, on the occurrence of harsh, dry, bronchial breathing over the root of the lungs posteriorly, and on a peculiar paroxysmal cough somewhat resembling that of pertussis.

**Nocturnal Enuresis.**—Dr. Donald MacAlister[13] writes strongly in support of the treatment of nocturnal enuresis in children by full doses of atropine. The formula he employs is as follows: ℞. liq. atrop. sulph. ʒiss., liq. strych. hydrochl. ♏xlv., syr. aurant. ad. ℥j. Sig. Five drops of the syrup to be taken in a tablespoonful of water at nine p.m. To be increased as directed. He considers that the addition of strychnine will counteract the depressant effects of large doses of atropine, and increase the sensitiveness of the vesical centres to reflexes from the bladder wall. Commencing with five drop doses of the above mixture, Dr. MacAlister increases the amount week by week until the patient is taking nightly fifty drops (about one-tenth grain of atropine). Physiological effects are, of course, produced, but cause only temporary inconvenience, and "the secret of success lies in courageous overdosing."

Pousson[14] states that incontinence of urine may arise from three different local conditions, namely—(1) Atony of the sphincter, (2) vesical irritability, and (3) irritability of the membranous portion of the urethra, or contracture of the sphincter. The two former are the most likely to benefit from treatment by belladonna. In all varieties medicines will sometimes fail to relieve, and recourse must then be had to local treatment by means of electricity. He employs the induced current, and applies it directly to the sphincter by means of a slender bougie containing a metallic conductor. After a few applications marked improvement usually follows.

At the Medical Society of Vienna, Professor von Hofmokl[15] showed a little girl whom he had successfully treated for incontinence of urine by Gersuny's method of twisting the urethra. Professor von Frisch considered that method preferable to any other local treatment.

**Infantile Paralysis.**—At the Medical Society of London Dr. Lewis Jones[16] read a paper on the electrical treatment of this disease. He recommended the continuous use of electricity for six months or a year in every case, as from experience he found it to be exceptional for a muscle to be so completely atrophied as to have no functional fibres left. He thought the treatment might be carried out by the mother or nurse by means of the induction coil. An interesting communication on the subject of acute anterior poliomyelitis has recently been published by Dr. Anna M. Galbraith,[17] in which she controverts many of the commonly accepted views. She believes that the disease is much more frequently fatal than is apparent from statistics, owing to the fact that it is often not correctly diagnosed in cases which terminate early in the illness. From her own experience of seventy-five cases it appears that a high fever of short duration, or a moderate fever lasting for a week or more, indicates an extensive and severe as well as permanent paralysis. In the acute stage she recommends leeches to the occiput and the ice bag to the spine, active purgation by means of calomel, and the rapid induction of diaphoresis. In the chronic stage, in addition to constitutional treatment, she advises the use of massage, stretching, electricity, and a suitable brace. In her own hands this treatment appears to have been unusually successful, and many interesting cases are recorded at length in the paper. In a clinical lecture on spasmodic infantile paralysis, Dr. F. Raymond[18] points out that syphilis plays a part in inducing these conditions. The affection may develop during fœtal life, or at the time of birth from traumatism, or in infancy. There is no definite pathological lesion for the various types of the disease, and the original morbid process cannot be deduced from the post-mortem changes.

**Peripheral Paralysis.**—Dr. William Gay[19] records a case following on varicella. The patient, aged two and a-half years, suddenly became completely paralysed in the lower extremities a fortnight after an attack of chicken-pox. For four weeks he was quite unable to move his legs. There was no evident wasting, but sensation was lost from the toes up to the middle of the thighs. The knee jerks and plantar reflexes were absent. At the end of three months he could walk fairly well, but the knee jerks were still absent. Dr. Gay discusses the alternative diagnoses in this case, and by a process of exclusion is led to conclude that the paralysis was dependent on the attack of varicella. Dr. J. A. Adams[20] has again drawn attention to the dangers accompanying the treatment of chorea with large doses of arsenic. The patient, a girl of eleven years, had been taking ten minims of Fowler's solution three times a day for three weeks, when symptoms of peripheral neuritis supervened, and in a few days she lost all power in the limbs. The arsenic was stopped, and she recovered gradually under suitable treatment. One point noted in these cases is that the neuritis develops without the occurrence of the cardinal symptoms of arsenical poisoning. A somewhat similar case is related by Dr. T. C. Railton[21], that of a girl of ten years who, after taking thirteen drachms of Fowler's solution in twenty-one days, developed motor paralysis, sensory disturbances, and ataxy. He considers that large doses, such as fifteen drops of the liquor arsenicalis, ought not to be continued longer than a week. Dr. W. R. Townsend[22] relates the case of a little girl suffering from diphtheritic paralysis affecting the neck muscles, who was sent to hospital with a diagnosis of Pott's disease. She had had an attack of diphtheria, and a few weeks later had fallen from her chair, and it was noticed that the head dropped forwards. Dr. Townsend had seen a number of similar cases in which the diphtheritic paralysis showed itself in the muscles of the neck, and pointed out that (1) the head could be lifted easily with the hand and placed in the normal position, and (2) the patient was able to support the head better in the morning than later on in the day, symptoms which served to distinguish this affection from Pott's disease.

[11] The Lancet, May 12th, 1894, p. 1,177. [12] Deut. Med. Wocheuschr, 1893, Nos. 9-17; and Am. Jour. Med. Sci., Dec., 1893. [13] Practitioner, May, 1894. [14] Journ. de Medec. de Bord., No. 21, 1893; and Am. Journ. Med. Sci., April, 1894. [15] The Med. Week., May 11th, 1894, p. 227. [16] B.M.J., March 10th, 1894, p. 525. [17] Am. Jour. of Obstet., Feb. and March, 1894. [18] Le Prog. Med., Vol. xix., No. 6; and Pract., April, 1894, p. 295. [19] B.M.J., March 31st, 1894, p. 679. [20] The Lancet, Feb. 10th, 1894. [21] B.M.J., Vol. ii., 1893, p. 996. [22] N. Y. Med. Jour., Feb. 24th, 1894

